# Debris Evaluation after Root Canal Shaping with Rotating and Reciprocating Single-File Systems

**DOI:** 10.3390/jfb7040028

**Published:** 2016-10-17

**Authors:** Alberto Dagna, Giulia Gastaldo, Riccardo Beltrami, Claudio Poggio

**Affiliations:** 1 Department of Clinical Surgical Diagnostic and Pediatric Sciences, Section of Dentistry, University of Pavia, Piazzale Golgi 3, Pavia 27100, Italy; alberto.dagna@unipv.it (A.D.); giuligast@gmail.com (G.G.); 2 Department of Brain and Behavioral Sciences, University of Pavia, Piazzale Golgi 3, Pavia 27100, Italy; rbreferee@gmail.com

**Keywords:** debris, NiTi, SEM, single-use instruments, single-file systems, smear layer

## Abstract

This study evaluated the root canal dentine surface by scanning electron microscope (SEM) after shaping with two reciprocating single-file NiTi systems and two rotating single-file NiTi systems, in order to verify the presence/absence of the smear layer and the presence/absence of open tubules along the walls of each sample; Forty-eight single-rooted teeth were divided into four groups and shaped with OneShape (OS), F6 SkyTaper (F6), WaveOne (WO) and Reciproc and irrigated using 5.25% NaOCl and 17% EDTA. Root canal walls were analyzed by SEM at a standard magnification of 2500×. The presence/absence of the smear layer and the presence/absence of open tubules at the coronal, middle, and apical third of each canal were estimated using a five-step scale for scores. Numeric data were analyzed using Kruskal-Wallis and Mann-Whitney U statistical tests and significance was predetermined at *P* < 0.05; The Kruskal-Wallis ANOVA for debris score showed significant differences among the NiTi systems (*P* < 0.05). The Mann-Whitney test confirmed that reciprocating systems presented significantly higher score values than rotating files. The same results were assessed considering the smear layer scores. ANOVA confirmed that the apical third of the canal maintained a higher quantity of debris and smear layer after preparation of all the samples; Single-use NiTi systems used in continuous rotation appeared to be more effective than reciprocating instruments in leaving clean walls. The reciprocating systems produced more debris and smear layer than rotating instruments.

## 1. Introduction

The aim of the root canal treatment is to shape root canals and to remove the pulp tissue, the bacteria and their byproducts [1,2,3,4,5]. Irrigating solutions promote the disinfection and the debridement of the endodontic space so they are necessary for the success of each root canal treatment [6,7,8,9,10]. Instruments alone are not able to eliminate bacteria and all modern nickel-titanium (NiTi) systems may produce a large amount of debris along the canal walls [11].

Several NiTi systems have been introduced in the market since they have been developed more than 20 years ago. Because of their motion inside the canal, they create debris and a smear layer that have to be removed with the aid of irrigating solutions [12,13]. Sodium hypochlorite (NaOCl) is a suitable bacteriostatic agent, but it is not effective in the removal of the inorganic amount of the smear layer [14,15,16,17,18,19]. Actually, the ideal way to favor the removal of organic debris and of the smear layer is to irrigate the canals with EDTA in combination with NaOCl [20,21,22]. EDTA is a chelating agent and it is able to decalcify the peritubular and the intertubular dentine, leaving the collagen exposed and the tubules opened. Thus, NaOCl can clean and disinfect the dentinal walls [16,17,18,19,20]. Each mechanical NiTi system used with irrigating solutions significantly decreases the microorganisms inside the root canals [21,22,23]. The removal of the smear layer helps the diffusion of the irrigating solutions inside the root canal space [11,12,13,14,15] and then favors the adhesion of obturation materials to dentine, reducing the apical and the coronal leakage [24].

Many efforts have been made to facilitate mechanical preparation of the endodontic space and to improve the predictability of each root canal treatment, so new NiTi instruments have been created to achieve this aim [23]. Single-use and single-file systems represent the most recent solution to make the root canal treatment easier (due to the reduction of the files necessary for complete root canal shaping) and safer (due to the reduction of stresses related to reuse, to disinfecting procedures and to thermal cycles in the autoclave). Single-use and single-file NiTi systems are available as reciprocating systems (WaveOne and Reciproc) and as rotating instruments (OneShape and F6 SkyTaper). They are single-file systems: only one file is required for the complete shaping of the root canal. They are single-use instruments: each file has to be discarded, not sterilized and not reused at the end of the treatment [25].

The purpose of this study was to investigate the dentinal surfaces of the root canals by SEM after shaping with rotating and with reciprocating single-file systems in order to evaluate the presence/absence of the smear layer and the presence/absence of open tubules at the coronal, middle, and apical third of each root canal. The aim is to compare the cleaning effectiveness of rotating and reciprocating systems.

## 2. Results

Table 1 and Table 2 report data derived from scoring for the presence/absence of debris and the smear layer. The Kruskal-Wallis ANOVA for debris score showed significant differences among the NiTi systems (*P* < 0.05). The Mann-Whitney U post-hoc test confirmed that WaveOne and Reciproc presented significantly higher score values than rotating NiTi systems (*P* < 0.05). Non-significant differences were obtained between WaveOne and Reciproc when considering scores of the debris and smear layer (*P* > 0.05), as reported in Table 3 and Table 4. For all NiTi systems, the ANOVA and Mann-Whitney test confirmed that the apical third of the canal maintains a higher quantity of debris and smear layer after preparation (*P* < 0.05), while the middle and coronal thirds present a lower amount of debris and smear layer (*P* > 0.05). A similar quantity of debris and smear layer was registered in canals shaped with OneShape and F6 SkyTaper (*P* > 0.05).

## 3. Discussion

Dentin debris and the smear layer are produced by the action of endodontic instruments during the root canal treatment and they are compacted along dentinal walls [3]. Their removal is essential because it could allow NaOCl to penetrate into the dentinal tubules and to improve its bactericidal action [7,8,9,10]. The presence of the smear layer along dentinal walls may reduce the adhesion of the sealers [4]. EDTA represents the gold standard for the removal of the smear layer formed during root canal shaping [26] and its association with NaOCl is effective for chemo-mechanical preparation. For this reason the irrigation technique with EDTA and NaOCl at each change of instrument was selected, as proposed by Foschi et al. [27].

NiTi instruments have evolved during the last 20 years: new designs and better alloys increase the shaping/cutting ability and resistance to fracture [28,29]. New generation files introduced innovative concepts such as reciprocating motion, single-file and single-use. All the tested instruments in this study are single-use: it means that they are not reused at the end of the treatment: no stresses are generated from chemical and thermal treatments and no stresses are related to previous root canal treatments. OneShape and F6 SkyTaper are single-file systems made for use in continuous rotation, WaveOne and Reciproc are single-file systems made for reciprocating motion. All instruments were evaluated in accordance with the manufacturer’s direction, respecting each operative protocol.

The continuous rotating systems showed better results than the reciprocating ones. They produced less debris and smear layer. Reciprocating motion have been shown to offer advantages in root canal preparation, but some doubts emerged regarding the accumulation of debris: De-Deus et al. [30] reported significantly larger debris accumulation after shaping with a traditional ProTaper F2 in reciprocating motion with respect to the same file used in continuous rotation. Robinson et al. [31] showed that a traditional rotary file is better than a reciprocating file in canals with isthmuses or lateral canals or recesses, because debris accumulation is lower. The same effects were obtained by Bürklein et al. [32] evaluating the apical extrusion of debris after root canal shaping with Reciproc, F360, OneShape, and MTwo: all instruments showed apical extrusion, but reciprocating instrumentation produced higher debris extrusion. It means that the continuous motion of the rotary files favors upward elimination of debris along the flutes of the file, while each backward motion of the reciprocating files compacts the debris along dentinal walls and pushes them into lateral canals and over the apex [31]. For this reason it is not a fault to believe that reciprocating files may work against themselves in extracting debris and dentinal chips from the root canal.

It is important to note that root canal anatomy is complex and in many cases the smear layer may accumulate in areas that are not touched by the instruments. Hard tissue debris accumulation is more frequent in isthmus areas, such as in the mesial roots of mandibular molars and in the mesiobuccal roots of maxillary molars [33]. As has been shown, accumulated debris certainly has a negative impact on the sealability of root canals, but it also may hamper disinfection in cases with apical periodontitis [33]. So, further investigations are needed to confirm or reject the results of the present study and to verify if reciprocating motion really works against itself by producing a bigger amount of debris along dentinal walls and not instrumented areas of the root canal system.

No significant differences emerged between the OneShape and F6 Skytaper systems. Even if a small amount of the smear layer is always visible, especially in the apical third, the main portion of the debris is eliminated when irrigation protocols with NaOCl and EDTA are respected.

## 4. Materials and Methods

Forty-eight single-rooted human teeth freshly extracted for periodontal reasons were selected for this study and placed in saline at room temperature immediately after extraction. The inclusion criteria were: morphological similarity, single-canal roots, straight roots, absence of root decay, absence of previous endodontic treatment, root length of at least 13 mm, and apical diameter of at least #20.

The crown of each tooth was removed at the level of the cementum-enamel junction (CEJ). Two longitudinal grooves were prepared along each root with a diamond bur to facilitate vertical splitting with a chisel after canal instrumentation.

All the roots were randomly assigned to four groups of 12 specimens each.

The root canals were preliminary scouted using stainless steel #10 K-files (MicroMega, Besancon, France) and then glide path was created with the 14.03 OneG single-use rotary Ni-Ti file (MicroMega, Besancon, France).

After preflaring, the samples were shaped with four different Ni-Ti rotary systems:

Group A: OneShape (MicroMega, Besancon, France),
Group B: F6 SkyTaper (Komet Brasseler GmbH & Co., Lemgo, Germany),
Group C: WaveOne (Dentsply Maillefer, Ballaigues, Switzerland),
Group D: Reciproc R25 (VDW, Munich, Germany).

OneShape (MicroMega, Besancon, France) and F6 SkyTaper (Komet, Brasseler GmbH & Co., Lemgo, Germany) were used in continuous rotation with the EndoMate DT motor (NSK, Kanuma, Japan). WaveOne (Dentsply Maillefer, Ballaigues, Switzerland) and Reciproc (VDW, Munich, Germany) were used in a reciprocating working motion generated by the VDW Silver Reciproc motor (VDW, Munich, Germany).

The root canals of Group A were prepared using the OneShape system (25.06) with speed set at 400 rpm and torque set 4.0 N/cm. The file was used reaching WL after three steps with gentle in-and-out motion.

The root canals of Group B were prepared using the F6 SkyTaper system (25.06) with speed set at 300 rpm and torque set 2.2 N/cm. The file was used reaching WL after three steps with gentle in-and-out motion.

The root canals of Group C were prepared using the WaveOne Primary (25.08) with the manufacturer configuration setup at the preset program “WaveOne All”. The WaveOne Primary instrument was used at working length (WL) with gentle in-and-out motion.

The root canals of Group D were prepared using the Reciproc R25 (25.08) with the manufacturer configuration setup at the preset program “Reciproc All”. The Reciproc R25 instrument was used at WL with gentle in-and-out motion.

For every group, the flutes of each instrument were cleaned frequently and root canals were irrigated with alternation of 1 mL of 5.25% NaOCl and of 1 mL of 17% EDTA. An equal amount of irrigating solution was used for each sample. At the end of preparation, 4 mL of 17% EDTA were left in situ for 120 s. followed by 1 mL of 5.25% NaOCl for 60 s. as a final rinse. Small 27G endodontic needless (Kendall Monoject, Mansfield, MA, USA) allowed to reach the apical third. At the end, all the canals were washed with ethanol for 30 s. and dried with calibrated paper points.

Each sample was dipped in liquid nitrogen immediately after canal preparation and split longitudinally into two halves with a stainless steel chisel. The sections were allowed to air-dry overnight in a desiccator at room temperature, sputter-coated with gold and prepared for SEM analysis (EVO MA 10, Carl Zeiss AG, Oberkochen, Germany).

**Figure 1:**
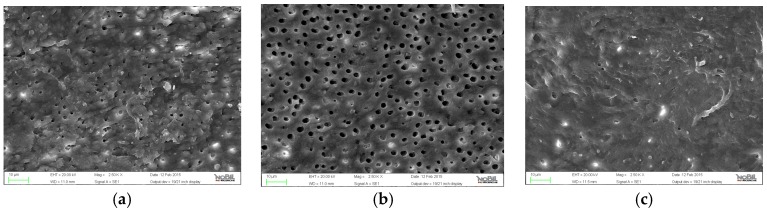
Representative samples of scanning electron micrographs of the root canal dentin surface instrumented with OneShape (group A) at coronal, middle and apical third of the root (magnification 2500×). (**a**) coronal third; (**b**) middle third; (**c**) apical third.

**Figure 2:**
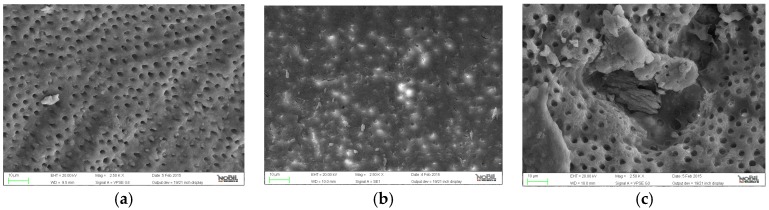
Representative samples of scanning electron micrographs of the root canal dentin surface instrumented with F6 SkyTaper (group B) at coronal, middle and apical third of the root (magnification 2500×). (**a**) coronal third; (**b**) middle third; (**c**) apical third.

**Figure 3:**
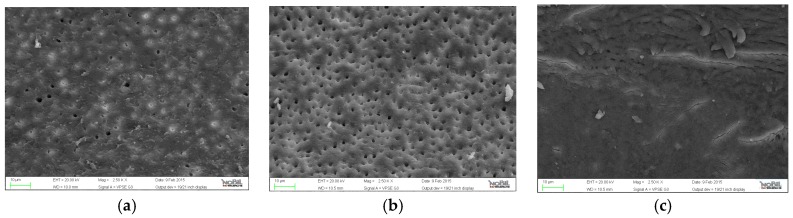
Representative samples of scanning electron micrographs of the root canal dentin surface instrumented with WaveOne (group C) at coronal, middle and apical third of the root (magnification 2500×). (**a**) coronal third; (**b**) middle third; (**c**) apical third.

**Figure 4:**
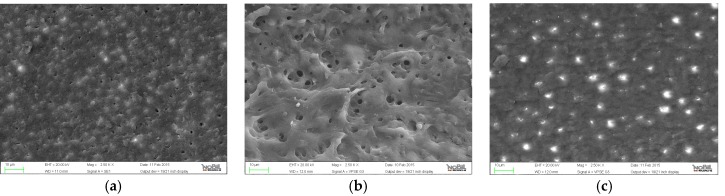
Representative samples of scanning electron micrographs of the root canal dentin surface instrumented with Reciproc (group D) at coronal, middle and apical third of the root (magnification 2500×). (**a**) coronal third; (**b**) middle third; (**c**) apical third.

SEM images were obtained at standard magnification of 2500× (Figure 1, Figure 2, Figure 3 and Figure 4). Six photomicrographs were taken at coronal, middle and apical third of the root canal. In a blind manner, three trained operators scored the presence or absence of debris and smear layer on the surface of the root canal at the coronal, middle, and apical portion of each canal. The rating system was proposed by Hulsmann et al. [24], and the criteria for the scoring are reported as follow:

Scores of the debris:

score 1: clean root canal walls, only few small debris particles,
score 2: few small agglomerations of debris,
score 3: many agglomeration of debris covering less than 50% of the root canal walls,
score 4: more than 50% of the root canal walls covered by debris,
score 5: complete or nearly complete root canal walls covered by debris.

Scores of the smear layer:

score 1: no smear layer, orifices of dentinal tubules open,
score 2: small amount of smear layer, some dentinal tubules open,
score 3: homogenous smear layer covering the root canal walls, only few dentinal tubules open,
score 4: complete root canal wall covered by a homogenous smear layer, no open dentinal tubules,
score 5: heavy, homogenous smear layer covering the entire root canal walls.

Statistical analysis was performed with Stata 12.0 software (Stata, College Station, TX, USA). Descriptive statistics for ordinal data, including the median, minimum and maximum values were calculated for all groups.

A non-parametric analysis of variance (Kruskal-Wallis ANOVA) and the post-hoc Bonferroni test were applied to investigate significant differences among treatments and among the three thirds of the canals. Significance for all statistical tests was predetermined at *P* < 0.05.

## 5. Conclusions

Within the limitations of this study, NiTi systems made for continuous rotation seem to be better than reciprocating instruments in obtaining clean canal walls, if irrigation protocols are respected (NaOCl + EDTA). The reciprocating systems (WaveOne and Reciproc) leave a higher quantity of debris and the smear layer is widely represented along root canal walls.

## Figures and Tables

**Table 1:** Summary score of the debris.

Group	Canal Level	Score = 1	Score = 2	Score = 3	Score = 4	Score = 5
**OS**	Coronal	8	4	0	0	0
	Middle	7	4	1	0	0
	Apical	8	2	2	0	0
**F6**	Coronal	6	4	1	1	0
	Middle	7	3	2	0	0
	Apical	3	4	4	1	0
**WO**	Coronal	2	5	4	1	0
	Middle	2	1	5	4	0
	Apical	1	2	6	2	1
**R25**	Coronal	3	3	5	1	0
	Middle	2	1	7	2	0
	Apical	2	0	8	2	0

**Table 2:** Summary score of the smear layer.

Group	Canal Level	Score = 1	Score = 2	Score = 3	Score = 4	Score = 5
**OS**	Coronal	7	4	1	0	0
	Middle	8	3	1	0	0
	Apical	7	4	0	1	0
**F6**	Coronal	6	2	2	2	0
	Middle	5	2	4	1	0
	Apical	5	4	2	1	0
**WO**	Coronal	4	4	4	0	0
	Middle	2	7	2	1	0
	Apical	0	3	5	3	1
**R25**	Coronal	4	6	1	1	0
	Middle	5	3	1	3	0
	Apical	2	6	2	2	0

**Table 3:** Kruskal-Wallis ANOVA performed among instrument groups and among canal thirds with data about the debris.

File	Debris					
	OS	F6	WO	Location	Apical	Coronal
F6	0.507	–	–	Coronal	0.005 *	–
WO	0.006 *	0.031 *	–	Middle	0.038 *	0.844
R25	0.019 *	0.033 *	0.911	–	–	–

Note: * significant differences.

**Table 4:** Kruskal-Wallis ANOVA performed among instrument groups and among canal thirds with data about the debris.

File	Smear Layer					
	OS	F6	WO	Location	Apical	Coronal
F6	0.632	–	–	Coronal	0.012 *	–
WO	0.015 *	0.045 *	–	Middle	0.029 *	0.789
R25	0.007 *	0.022 *	882	–	–	–

Note: * significant differences.
